# Solid-State Characterization and Biological Activity of Betulonic Acid Derivatives

**DOI:** 10.3390/molecules201219876

**Published:** 2015-12-18

**Authors:** Ionuţ Ledeţi, Ştefana Avram, Vasile Bercean, Gabriela Vlase, Titus Vlase, Adriana Ledeţi, Istvan Zupko, Marius Mioc, Lenuţa-Maria Şuta, Codruţa Şoica, Cristina Dehelean

**Affiliations:** 1 Faculty of Pharmacy, Victor Babeş University of Medicine and Pharmacy, 2 Eftimie Murgu, 300041 Timisoara, Romania; ionut.ledeti@umft.ro (I.L.); marius.mioc@umft.ro (M.M.); suta.lenuta@umft.ro (L.-M.S.); codrutasoica@umft.ro (C.S.); cadehelean@umft.ro (C.D.); 2 Faculty of Industrial Chemistry and Environmental Engineering, Politehnica University Timişoara, 6 Carol Telbisz, 300001 Timisoara, Romania; 3 Research Centre for Thermal Analysis in Environmental Problems, West University of Timişoara, 300115 Timisoara, Romania; gabriela.vlase@e-uvt.ro (G.V.); titus.vlase@e-uvt.ro (T.V.); 4 Department of Pharmacodynamics and Biopharmacy, University of Szeged, 6 Eotvos Str., H-6720 Szeged, Hungary; zupko@pharm.u-szeged.hu

**Keywords:** betulonic acid derivatives, triterpene, thermal analysis, synthesis, biological activity, MTT assay

## Abstract

Betulonic acid belongs to the pentacyclic triterpenic derivative class and can be obtained through the selective oxidation of betulin. In this study we set obtaining several functionalized derivatives of this compound by its condensation with several amino compounds such as aminoguanidine, hydroxylamine, *n*-butylamine and thiosemicarbazide as our goal. The functionalization of the parent compound led to several molecules with antiproliferative potential, the most promising being 3–2-carbamothioylhydrazonolup-20(29)-en-28-oic acid.

## 1. Introduction

Naturally occurring compounds have always been an important source of pharmacologically-active molecules, associated with numerous biological targets and receptors. The importance of studying naturally occurring compounds is also sustained by the fact that most plants are accessible resources, the main problems consisting only in separation and purification of the bioactive derivatives contained therein, which can be realized by employing numerous different instrumental and experimental techniques [1,2,3]. Among all naturally occurring compounds, triterpenoids have been intensely studied in the last decades, since these compounds are widely distributed in numerous plants and possess important biological activities [4,5,6,7,8,9].

Betulin-skeleton compounds (including acids, aldehydes and their functionalized derivatives) are well studied and recent references for numerous biological activities are reported [10,11], such as treatment of liver fibrosis [12], proapoptotic and inhibition of cell migration in cancer [13,14], antitumour [15,16], anti-mycobacterial [17], hypoglycemic [18], effective adjuvant treatment option in pruritic dry skin [19], antiviral effect [20] and also immunomodulatory [21] and anti-inflammatory activities [22].

**Figure 1:**
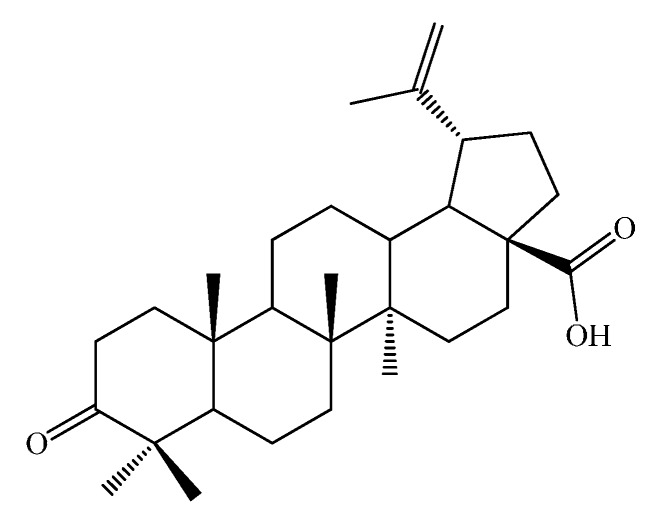
Structural formula of betulonic acid (BetO).

In the field of functionalization and evaluation of biological activities, betulonic acid and its derivatives are by far less studied and reported in literature compared to other triterpenoids. Betulonic acid (or lup-20(29)-en-3-oxo-28-oic, BetO, Figure 1)) and some of its derivatives were previously evaluated for biological activities, showing antiviral [23,24], antitumor and antineoplastic [25,26], hepatoprotective [27], anti-inflammatory [28,29], anti-HCMV activity [30], antimicrobial [31] and immunotropic effects [32].

The synthesis and functionalization of betulonic acid was previously reported in the literature by some authors, mainly focusing on physico-chemical investigation results, but some correlations with biological activities are available as well [29,33,34,35]. With this consideration in mind, we set as our goal the physico-chemical characterization and a preliminary evaluation of the biological activity of four derivatives obtained by functionalization of betulonic acid at position 3 via condensation with amino compounds. Compound **C2** was previously reported by Flekhter *et al.* in 2004 [34] and later by Genet *et al.* in 2010 [36]. The derivative **C1** was quite recently evaluated as an inhibitor of osteoclast differentiation [37].

## 2. Results and Discussion

### 2.1. Synthesis

The syntheses of the functionalized derivatives of betulonic acid were carried out in anhydrous organic solvents like pyridine (**C1**, **C2**), toluene (**C3**) or ethanol (**C4**), using as starting material betulonic acid obtained from betulin by a modified Jones oxidation method, previously reported, along with the spectroscopic data and purity, by Ledeti and Bercean [33]. The functionalization of BetO was carried out in the 3-oxo position by condensation with several amino compounds, like aminoguanidine, hydroxylamine, n-butylamine and thiosemicarbazide, used in molar ratios ranging from 1:1 up to 1:2.

### 2.2. Characterization of Compounds

#### 2.2.1. Physico-Chemical Data

The NMR data for the starting material used (BetO) was previously reported by or our group [33], proving the purity of the starting compound. The physico-chemical characteristics of each compound are presented in Table 1 (Experimental Section).

**Figure 2:**
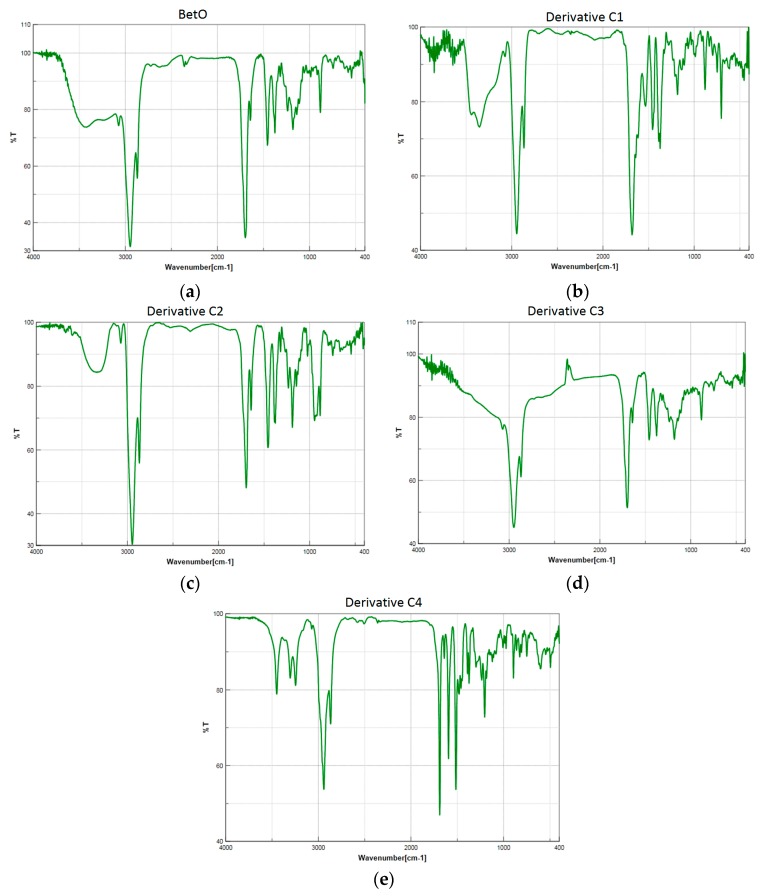
FTIR spectra of the analyzed compounds: (**a**) starting material BetO; (**b**) derivative **C1**; (**c**) derivative **C2**; (**d**) derivative **C3**; (**e**) derivative **C4**.

The spectra of these triterpenic substances are very similar (Figure 2). For all compounds, the strong band in the 1712–1690 cm^−1^ range is identified as the ν^as^(COO) vibration . The band assigned to the vibration of ν^s^(COO) is observed in the 1460–1385 cm^−1^ range. In the case of **C1** and **C2**, the symmetrical carboxyl group vibrations are split into two peaks. In the IR spectra of these compounds, several vibrations characteristic of the triterpenic moieties are also present. The characteristic bands attributed to the C=N stretching vibration are found at 1642 cm^−1^ for compounds **C1**–**C3**, respectively, and 1640 cm^−1^ for derivative **C4**, and this is in good agreement with the data mentioned in the literature (between 1610 and 1665 cm^−1^) [38].

In compound **C2**, the oxime functional group (C=N-OH) is represented by the vibrations at ν = 3300 cm^−1^ for the O-H stretch; 1642 cm^−1^ for the C=N stretch and 945 cm^−1^ for the N-O bond, respectively. For compound **C1**, the amino group is evidenced by the two stretching vibrations peaks found in the 3400–3300 cm^−1^ range. The N=C-N stretching vibration peak appears at 1374 cm^−1^, and the sharp bands in the 1160–1110 cm^−1^ interval correspond to the N-N stretching of the hydrazinic part of the aminoguanidine moiety [39]. These bands confirm the functionalization of the betulonic acid, and the results are also correlated with the ones obtained by thermal analysis.

#### 2.2.2. Thermal Analysis of Decomposition in Air

**Figure 3:**
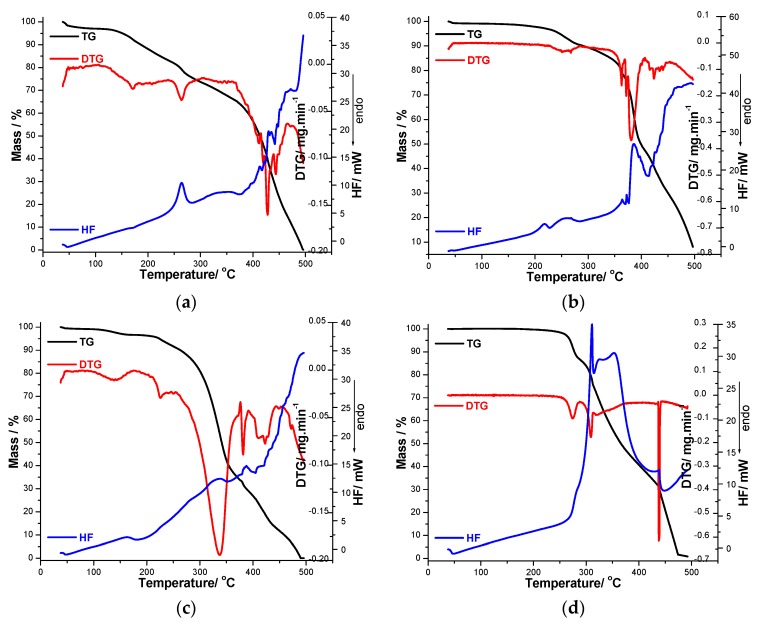
Thermoanalytical TG/DTG/HF data recorded in air atmosphere up to 500 °C for compounds (**a**) **C1**; (**b**) **C2**; (**c**) **C3** and (**d**) **C4**.

TG/DTG/HF analysis was used to describe the thermal stability and degradation of the synthesized compounds in an oxidative air atmosphere. The first mass loss step, occurring at low temperatures, is dehydration. After that, partial decomposition and the total decomposition of the organic structure take place. Most processes are poorly separated from each other, since the decomposition is clearly multistage, with overlapping processes. The thermal properties of the synthesized triterpenic compounds were evaluated by TG, DTG and HF. TG/DTG/HF curves providing information about the thermal degradation of the compounds are shown in Figure 3a–d. The thermoanalytical curves vary in all four cases.

In the case of derivative **C1**, the water elimination takes place in the temperature range 57–184 °C, with a maximum on the DTG curve at 170 °C and Δm = 9.7%. The next thermal process defines the melting of the compound, which takes place with decomposition (Δm = 16.8%; DTG_max_ = 263 °C and HF_max_ = 264 °C). This melting temperature in good agreement with the value obtained using the Boetius apparatus (T_melting_ = 274–277 °C).

After that, an intensive thermodegradation takes place, with a 73.3% mass loss and characterized by several peaks on the DTG curve at 410, 419, 428 and 444 °C, respectively. The HF curve has a strong upward slope defining a strongly exothermic effect which accompanies the thermal degradation of the triterpenic structure (Figure 3a).

In the case of the functionalized compound **C2**, the mass loss starts from ~118 °C, according to the thermogravimetric curve. After recrystallization, the observed melting point is in good agreement with the one mentioned in literature (223–225 °C) [34]. Melting (acc. to Boetius between 225–229 °C) is also revealed by the thermoanalytical technique, and takes place with decomposition (HFmax = 228 °C, Δm = 9.8%). In the next temperature range, derivative **C2** showed a pyrolitic decomposition involving more successive processes. In the temperature range of 287 to 406 °C, there was a 42.7% mass loss and between 406 and 500 °C, the mass loss was 39.5%, leading to a final mass of approx. 0% (Figure 3b).

As shown in Figure 3c, the thermal decomposition of **C3** takes place in five degradation stages. The first mass loss corresponds to a dehydration process. The water loss takes place in the temperature range of 95–176 °C and corresponds to a mass loss of 3.4%. After this process, an endothermic peak appears on the HF curve at 183 °C, highlighting the melting (no mass loss is observed on the TG curve). The total decomposition of **C3** starts above 200°. After water release, the fragmentation of the triterpenic compound starts. The first mass loss after melting represents 5.5% and is characterized by a sharp peak on DTG curve with maximum at 224 °C. In the temperature range of the degradation process (203–500 °C), several exothermal processes are revealed by the analysis of the HF curve. Above 333 °C, the decomposition of this compound is highly exothermic and the final degradation occurs at 488 °C, leaving a residue mass of ~0.05%.

The fourth analyzed compound (**C4**) is the most stable, with its decomposition starting at 245 °C. The first step in the TG curve ends at 289 °C with a mass loss of 14% and corresponds with a defined peak on the DTG curve with a maximum at 274 °C. The mass loss process is a continous one until 500 °C when the complete mass loss took place. The steps can be separated only on the basis of the DTG curve. The second step begins at 289 and ends at 315 °C with a mass loss of 11% and it is assigned to the oxydation of the triterpenic structure of the compound being accompanied by an strong exothermic peak at 312 °C (exothermic combustion). Finally, for the last stage of degradation (315–500 °C range), it can be assumed that the entire structure of the analyzed compound is totally oxidized and eliminated as volatile compounds leaving a small residue with Δm ≈ 0.1% (Figure 3d).

### 2.3. Biological Activities

**Figure 4:**
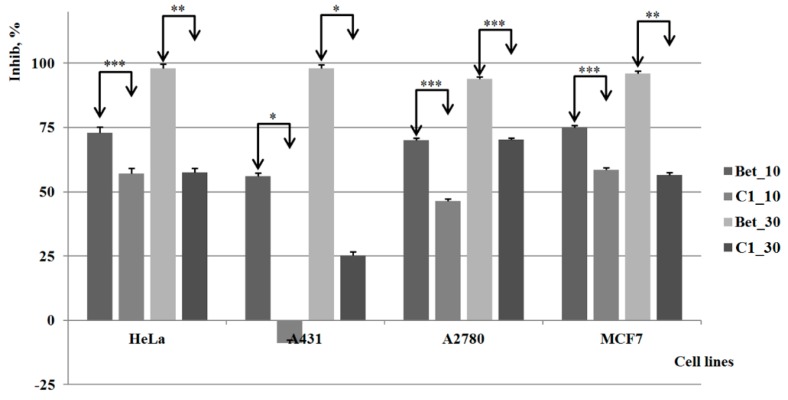
The results of the *in vitro* tests for **C1** *vs.* control group (Bet) *, **, *** indicate *p* < 0.05, *p* < 0.01, and *p* < 0.001.

**Figure 5:**
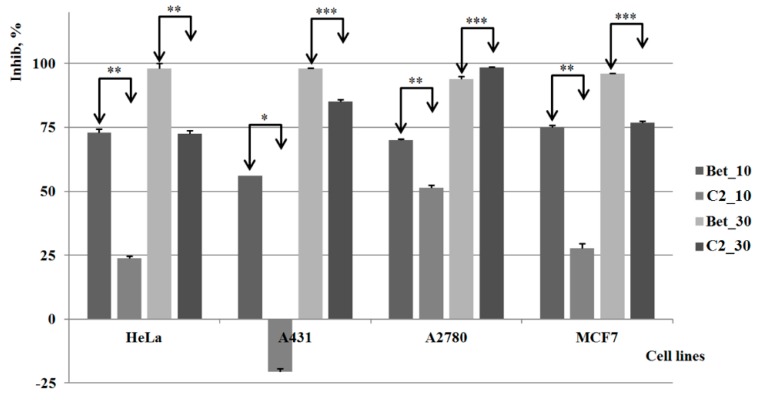
The results of the *in vitro* tests for **C2** *vs.* control group (Bet) *, **, *** indicate *p* < 0.05, *p* < 0.01, and *p* < 0.001.

**Figure 6:**
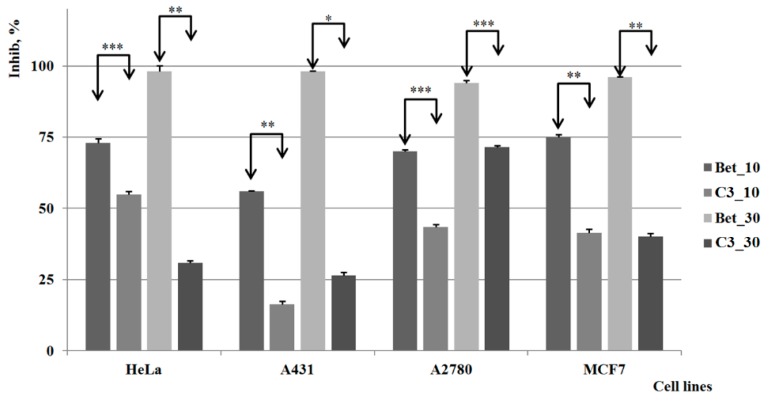
The results of the *in vitro* tests for **C3** *vs.* control group (Bet) *, **, *** indicate *p* < 0.05, *p* < 0.01, and *p* < 0.001.

**Figure 7:**
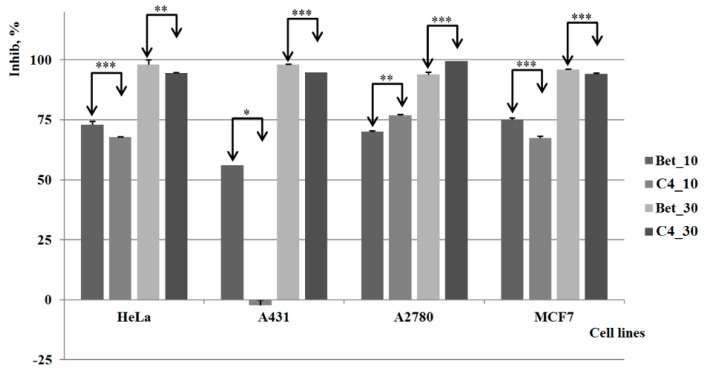
The results of the *in vitro* tests for **C4** *vs.* control group (Bet) *, **, *** indicate *p* < 0.05, *p* < 0.01, and *p* < 0.001.

The results of the *in vitro* tests are displayed in Figure 4, Figure 5, Figure 6 and Figure 7; all results were graphically presented as comparisons with a known antiproliferative compound of similar structure, betulin. The analysis was conducted in two concentrations as described in previous studies [40].

All four tested compounds are chemical derivatives obtained by the modulation of the C-3 carbonyl group of betulonic acid, a triterpene compound proven as active against several tumor cell lines, such as MGC-803–human gastric cancer, PC3–prostatic cancer and MCF7–breast adenocarcinoma [41]. The related compounds, betulin and betulinic acid were thoroughly studied by our research group, which revealed the important antiproliferative activities on the A431, MCF7 and HeLa tumor cell lines [42]; also, significant *in vitro* antitumor effects were reported against other various cell lines [43,44]. In addition, betulin (Bet) was evaluated as antiangiogenic agent on the same three cell lines, A431, HeLa and MCF7, in a study that also involved establishing the dose-effect relationship [42]. The results showed a high specificity of betulin against cervical (HeLa) and skin (A431) cancer accompanied by a significant activity against breast adenocarcinoma (MCF7). Based on previous findings, betulin (Bet) was used as positive control in the current study which also involves three previously studied tumor cell lines.

Derivative **C1** revealed weaker cytotoxic effects on all four cell lines as compared to pure betulin, regardless of the concentration involved (Figure 4). Moreover, the cytotoxic activity is completely absent on the A431 cell line when the lower concentration was used, and even at higher concentration the inhibitory activity against this cancer cell line of **C1** was insignificant. On both HeLa and MCF7 cell lines, the cell viability was significantly reduced in a concentration-independent manner, thus revealing an external limitation of the anticancer effect of **C1**, presumably its poor water solubility. A dose-activity relationship was noticed on the A2780 cell line; however, the results reveal weaker inhibitory activity as compared to pure betulin.

Compound **C2** revealed a strong cytotoxic activity comparable to betulin on the A2780 cell line when used at the lower concentration, and even higher than betulin when the higher concentration was used; the compound also causes a significant inhibitory process against the HeLa and MCF7 cell lines (Figure 5). By contrast, the cytotoxic activity is practically insignificant on A431 when the lower concentration is applied; a strong inhibitory activity can be noticed however for higher concentrations. Therefore, one can state that this compound possesses an important antitumoral/cytotoxic effect on certain tumor cell lines, highly dependent on its concentration.

Compound **C3** displayed a quantifiable inhibitory activity against all tested cell lines; however, the results are not very promising in comparison to pure betulin, which shows stronger cytotoxic activity at both concentrations. As depicted in Figure 6, the cytotoxic effect of both compounds is developed in a dose-relationship manner. The highest activity manifested by the compounds’ lower concentrations occurs on HeLa cells, in contrast to the higher concentrations that were relevant against ovarian carcinoma (A2780). For the MCF-7 (breast carcinoma) cell line the results show a similar activity for both concentrations, comparable to the one noticed against ovarian carcinoma (A2780).

Compound **C4** revealed significant cytotoxic effects against all types of cell lines involved in the study; at the lower concentration, it proved to have comparable inhibitory activity to pure betulin on the HeLa and MCF7 cell lines, while no activity was noticed against the A431 cell line. The A2780 cell line proved to be the most sensitive to this compound, the inhibition percentage reaching 100% for the higher concentration while at the lower concentration, the compound remains more active than pure betulin (Figure 7).

The results show that the chemical functionalization of the parent compound led to several molecules with cytotoxic potential, the most promising being **C4**; we may assume that the water solubility of the tested compounds is an important factor to be considered in future studies due to the large variations noticed in terms of *in vitro* activity.

## 3. Experimental Section

### 3.1. General Information

All reagents and solvents were commercial products of Merck (Darmstadt, Germany) and used without further purification. Betulonic acid used as starting material was previously prepared and characterized by appropriate instrumental techniques in our laboratory, and the results were already published [33]. Reduced-pressure evaporation of solvents was realized using a R-200 rotary evaporator (Büchi, Flawil, Switzerland) equipped with a Büchi heating bath B-490 and coupled to a Rotavac vacuum pump. Thin-layer chromatography (TLC) was performed with silica gel-coated plates 60 F_254_ (Merck) using hexane–ethyl acetate 6:4 (*v*:*v*) and hexane–ethyl acetate 1:1 (*v*:*v*) as eluants. The chromatographic spots were revealed by exposure to a mixture of H_2_SO_4_ 5%, ammonium wolframate 5% and cerium sulphate 0.2% and/or UV light irradiation (λ = 254 nm). Melting points were determined on a Böetius PHMK (Veb Analytik, Dresden, Germany) instrument and are uncorrected. The percentages of C, H and N were obtained by means of elemental analysis using a El Cube apparatus (Vario, Hanau, Germany). The thermal stabilities of the synthetized compounds were determined on a DIAMOND thermo-balance (Perkin Elmer, Waltham, MA, USA) by recording the corresponding thermoanalytical TG, DTG and HF curves. The samples (weighing between 6.2 and 8.6 mg) were heated in aluminum crucible between 25 and 500 °C in a dynamic air atmosphere (100 mL·min^−1^) with a heating rate β of 10 °C·min^−1^. For determining the thermal effects, the DTA data (in µV) were converted to HF (Heat Flow) data (mW). In order to assure the reproducibility of the TG study, each analysis was repeated three times. The results were practically identical. FTIR spectra were recorded on a JASCO FTIR/670plus device (Jasco, Easton, MD, USA) using the KBr pellet dispersion method. The data was collected in the 4000–400 cm^−1^ range. Spectra were built up after 36 acquisitions for each spectrum.

### 3.2. Synthesis and Characterization

All the syntheses started from betulonic acid (*BetO*), which was dissolved in the minimum amount of solvent (anhydrous pyridine for **C1** and **C2**, absolute ethanol for **C4**), then treated with aminoguanidine hydrogen carbonate (AmgH, for **C1**), hydroxylamine hydrochloride (HAH, for **C2**), or thiosemicarbazide (TSC, for **C4**). The mixtures were refluxed (**C1** and **C4**) or stirred at room temperature (**C2**). After distilation under reduced pressure, the derivatives were purified by recrystallisation of the crude product from ethanol (**C1**, **C4**) or previously dissolved in chloroform and washed with water (**C2**). Derivative **C3** was obtained in cold (0 °C) anhydrous toluene under inert gas, by treating a mixture of *n*-butylamine (nBuA) with titanium (IV) chloride (TiCl_4_) in a 5.3:1 nBuA:TiCl_4_ molar ratio, with BetO (molar ratio TiCl_4_:BetO 1:0.75). After heating under reflux for 7 h and distilling to dryness under reduced pressure, the crude compound was suspended in ethyl acetate, washed twice with 15% HCl, then distilled water and dried over anhydrous Na_2_SO_4_. The crude compound was recrystallized from ethanol. The physico-chemical characteristics of **C1**–**C4** are listed in Table 1.

**Table 1:** The physico-chemical characteristics of the obtained functionalized derivatives **C1**–**C4**.

Structure	Analytical Data
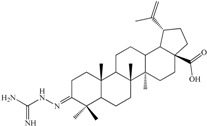	3-2-Carbamimidoylhydrazonolup-20(29)-en-28-oic acid (**C1**). White crystalline solid (yield η = 66.7%), m.p. (Boetius) = 274–277 °C; FTIR (KBr, cm^−1^): 3442, 3352, 3175, 3070, 2946, 2867, 1680, 1642, 1457, 1537, 1455, 1375, 1184, 989, 882, 749, 703, 617. Elemental Analysis (Calcd./Found) for C_31_H_50_N_4_O_2_, M = 510.75 g/mol: C = 72.90%, H = 9.87%, N = 10.97%; Found: C = 73.04%, H = 9.91%, N = 11.07%
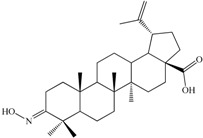	3-Hydroxyiminolup-20(29)-en-28-oic acid (**C2**). White crystalline solid (yield η = 72.4%), m.p. (Boetius) = 210 °C (phase transition); 219–224 °C (crude); m.p.(Boetius) = 225–229 °C (EtOH); FTIR (KBr, cm^−1^): 3283, 3073, 2946, 2869, 1697, 1642, 1455, 1385, 1376, 1320, 1267, 1233, 1188, 1144, 1023, 945, 885, 795, 746, 665, 567, 544, 501; Elemental Analysis (Calcd./Found) for C_30_H_47_NO_3_, M = 469.70 g/mol: C = 76.71%, H = 10.09%, N = 2.98%; Found: C = 76.15%, H = 10.23%, N = 2.64%
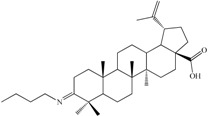	3-Butyliminolup-20(29)-en-28-oic acid (**C3**). Pale yellow solid (yield η = 73.4%), m.p. (Boetius) = 145–150 °C; FTIR (KBr, cm^−1^): 3072, 2986, 2940, 2868, 1694, 1642, 1454, 1375, 1279, 1233, 1180, 1110, 1024, 882, 744, 543; Elemental Analysis (Calcd./Found) for C_34_H_55_NO_2_, M = 509.81 g/mol: C = 80.10%, H = 10.87%, N = 2.75%; Found: C = 80.98%, H = 11.29%, N = 2.82%
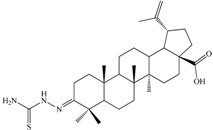	3-(2-Carbamothioylhydrazono)lup-20(29)-en-28-oic acid (**C4**). White solid (yield η = 28.1%), m.p.(Boetius) >330 °C; FTIR (KBr, cm^−1^): 3449, 3303, 3245, 2940, 2867, 1690, 1640, 1515, 1481, 1458, 1389, 1373, 1236, 1204, 973, 894, 602, 495; Elemental Analysis (Calcd./Found) for C_31_H_49_N_3_O_2_S, M = 527.80 g/mol: C = 70.54%, H = 9.36%, N = 7.96%; Found: C = 70.15%, H = 9.59%, N = 8.01%

### 3.3. Biological Activity

The compounds’ cytotoxic effects were evaluated using a MTT ([3-(4,5-dimethylthiazol-2-yl)-2,5-diphenyltetrazolium bromide]) colorimetric assay and was performed on four human cell lines (obtained from ECACC; Salisbury, UK): HeLa (cervix adenocarcinoma), MCF7 (breast adenocarcinoma), A431 (skin epidermoid carcinoma) and A2780 (human ovarian carcinoma) [45]. The cells were cultivated by applying minimal essential medium supplemented with 10% fetal bovine serum, 1% non-essential amino acids and an antibiotic/antimycotic mixture. All media and supplements were obtained from PAA Laboratories (Pasching, Austria). The cells were grown in a humidified atmosphere of 5% CO_2_ at 37 °C. Cancer cells (5000/well) were seeded onto a 96-well microplate and attached to the bottom of the well overnight. On the second day, 200 μL of new medium containing the test substances were added. After 72 h incubation, the living cells were assayed by the addition of 20 μL of 5 mg/mL MTT solution. MTT was converted by the intact mitochondrial reductase and precipitated as its formazan crystals during a 4 h contact period. The medium was then removed, and the precipitated crystals were dissolved in 100 μL dimethyl sulfoxide (DMSO) under stirring for 60 min. Finally, the reduced MTT was spectrophotometrically analyzed at 545 nm, using a microplate reader; wells with untreated cells were used as controls. All *in vitro* experiments were carried out on two independent microplates with at least five parallel wells; the entire MTT evaluation was conducted under the same conditions for all samples (time, preparation method). Stock solutions of the tested substances (10 mM) were prepared using DMSO as solvent; the highest DMSO concentration (0.3%) was proven to not have any significant effect on cell proliferation.

### 3.4. Statistical Analysis 

Paired Student’s *t* tests or One-way ANOVA were used to determine the statistical differences between various experimental and control groups.

## 4. Conclusions

In this paper, we presented the functionalization of betulonic acid, which is a semi-synthetic derivative obtained by selective oxidation of the naturally abundantly occurring betulin. The functionalization of betulonic acid was carried out at the 3-oxo position and aimed at the condensation with several amino compounds like aminoguanidine, hydroxylamine, *n*-butylamine and thiosemicarbazide, used in molar ratios from 1:1 up to 1:2. All the compounds were characterized by physico-chemical tools, including elemental analysis, FTIR spectroscopy and thermogravimetry and later assayed for the cytotoxic activity. The *in vitro* biological evaluation led to the conclusion that the most promising compound is 3-(2-carbamothioylhydrazono)-lup-20(29)-en-28-oic acid (**C4**).
